# Sex Differences in White Matter Pathways Related to Language Ability

**DOI:** 10.3389/fnins.2019.00898

**Published:** 2019-08-28

**Authors:** Minyoung Jung, Maria Mody, Toru Fujioka, Yukari Kimura, Hidehiko Okazawa, Hirotaka Kosaka

**Affiliations:** ^1^Department of Neuropsychiatry, University of Fukui, Eiheiji, Japan; ^2^Biomedical Imaging Research Center, University of Fukui, Eiheiji, Japan; ^3^Research Center for Child Mental Development, University of Fukui, Eiheiji, Japan; ^4^Athinoula A. Martinos Center for Biomedical Imaging, Massachusetts General Hospital, Harvard Medical School, Boston, MA, United States; ^5^Special Needs Education Subcourse, Primary Education Course, School of Education, University of Fukui, Eiheiji, Japan

**Keywords:** white matter pathways, language, sex difference, cingulum-cingulate gyrus supracallosal bundle, corticospinal tract, corpus callosum, forceps minor

## Abstract

Evidence from functional imaging studies points to a role for gender in language ability. However, recent studies suggest that sex differences in the neural basis of language are still unclear, reflecting a complex interaction between sex and language ability. We used diffusion weighted magnetic resonance imaging and global probabilistic tractography to investigate white matter (WM) pathways between 32 male and 35 age- and IQ-matched female adult participants in relation to their verbal abilities. Males showed higher fractional anisotropy (FA) in the left anterior thalamic radiations (ATR), right cingulum-angular bundle, right corticospinal tract, bilateral superior longitudinal fasciculus-temporal terminations, bilateral uncinate fasciculus (UNC), and corpus callosum-forceps minor when compared with the female group. In contrast, females showed higher radial diffusivity (RD) in the left ATR and left UNC when compared to the male group. The relationship between WM metrics and verbal ability also differed across the two groups: a negative correlation between verbal comprehension index (VCI) and FA as well as axial diffusivity (AD) in left cingulum-cingulate gyrus (CCG) supracallosal bundle in males but not in females; a negative correlation between verbal IQ (VIQ) and FA in the right corticospinal tract (CST), and a positive correlation between VCI and RD in corpus callosum-forceps minor in the female but not in the male group. A direct comparison of these correlation coefficients yielded significant differences between the groups for the VCI-AD and VIQ -FA associations. The findings may reflect sex differences in WM related to language ability.

## Introduction

Over the past two decades, neuroimaging studies have significantly contributed to our understanding of the relationship between brain structure and language processes (Geschwind, 1965; Glasser and Rilling, 2008; Friederici, 2009). Traditionally, Broca’s area and Wernicke’s area have been considered to play central roles in language production and comprehension, respectively (Geschwind, 1965; Goodglass et al., 1972). However, recent neuroimaging studies using diffusion weighted imaging (DWI) have begun to shed light on the anatomical and functional connections between these brain areas mediated via white matter (WM) pathways that are also associated with language ability (Burman et al., 2008; Glasser and Rilling, 2008; Friederici, 2009; Wilson et al., 2011). For example, the superior longitudinal fasciculus (SLF) which connects frontal and opercularis regions that make up Broca’s area with temporo-parietal brain regions, is associated with comprehension and production of language (Catani and Thiebaut de Schotten, 2008; Bernal and Altman, 2010; Wilson et al., 2011). A separate pathway, the cingulum-cingulate gyrus supracallosal bundle (CCG) which connects the cingulate with other brain regions, has also been associated with language, specifically phonological processing (Catani and Thiebaut de Schotten, 2008; Walton et al., 2018).

Early language research for the most part has focused on documenting the more universal processes of language acquisition, such as vocabulary and speech sound development. However, variations in the pattern or time course of acquisition provide a window into the underlying mechanisms of language (Bates et al., 1995), and the sex of the child has been repeatedly pointed to as a source of inter-individual variations in language ability. The findings, though, have not been consistent, and are influenced by age, socioeconomic status and language domain, among other variables (Barbu et al., 2015). Mehl et al. (2007) found that women and men produce about the same amount of spontaneous speech in everyday life interactions. A meta-analytic study also revealed that verbal abilities of men and women are roughly comparable (Hyde and Linn, 1988). On the other hand, findings suggest that females acquire words faster than males (Roulstone et al., 2002), reflecting a difference in language learning strategies between the two sexes (Ehrman and Oxford, 1989); furthermore, males show greater use of spatial language than females (Pruden and Levine, 2017), whereas females produce more spontaneous language (Craig and Washington, 2002). Sex differences have also been found in task-based functional MRI studies (Shaywitz et al., 1995; Clements et al., 2006; Burman et al., 2008; Fonagy et al., 2012; Yu et al., 2014; Etchell et al., 2018; see Ihnen et al., 2009 and reviews by Kaiser et al. (2009), Wallentin (2009) for absence of gender effects). For instance, females showed greater neural activity than males in the inferior frontal gyrus and superior temporal gyrus in spelling and rhyming tasks (Burman et al., 2008) whereas males showed greater left frontal activation in semantic retrieval tasks (Konrad et al., 2008). Males also presented with more left lateralized activity during verb generation; females, in contrast, showed a more bilateral pattern of brain activation (Yu et al., 2014).

Given the growing recognition of the role of connectivity in brain function, we focus here on WM connections. There is general consensus that males and females show differences in language activation patterns in the brain; however, sex differences in WM pathways with regard to language ability remain unclear. In the current study, we examined fractional anisotropy (FA), a measure of WM microstructural integrity (Wheeler-Kingshott and Cercignani, 2009), mean diffusivity (MD) which reflects the magnitude of randomized water motion (Song et al., 2005), axial diffusivity (AD) for axonal integrity (Karahanoğlu et al., 2018), and radial diffusivity (RD) for myelin integrity (Song et al., 2005) to enrich our understanding of quantitative WM differences. The aim of the current study was to identify WM pathways associated with language ability in healthy adults, males and females, related to language ability. We hypothesized that there would be differences in the relationship between WM pathways and language ability associated with sex.

## Materials and Methods

### Participants

Healthy adult participants, males and females, were recruited via flyers posted around Fukui prefecture in Japan and advertisements posted on campus at the University of Fukui. The exclusion criteria for this study were: (1) history of brain injury, history of major medical conditions, or a lifetime history of alcohol or drug dependence, (2) current use of psychiatric medication, (3) left-handedness [assessed by the Edinburgh handedness inventory (Oldfield, 1971)] to avoid potential issues related to language lateralization, (4) Full Scale IQ (FSIQ) less than 85. Seventy-two participants underwent T1-weighted MRI and DWI. Data from four participants were excluded from the analysis because of poor MR and/or diffusion image quality based on previous study guidelines: T1 and DTI images were rated for quality using a six-point Likert scale (unusable, poor, fair, good, very good, and excellent) and excluded if an image was rated unusable, poor, or fair (Backhausen et al., 2016). The sample in the final analysis consisted of a total of sixty-seven healthy adults, 32 males (mean age: 24.1 years, s.d.: 4.1) and 35 females (mean age: 24.9 years, s.d.: 5.4), with no significant age difference between the groups (*p* = 0.53, n.s.). Participants had normal hearing and normal or corrected-to-normal vision or no history of sex change. The study was approved by the Institutional Review Board of the University of Fukui and all participants gave written informed consent.

### Language Ability Measures

The Wechsler Adult Intelligence Scale (WAIS-III; Wechsler, 1997) was used to investigate language and other performance abilities of the participants based on a previous study design (Conti-Ramsden et al., 2007). Measures included full scale IQ (FSIQ), verbal IQ (VIQ), performance IQ (PIQ), verbal comprehension index (VCI), working memory index (WMI), perceptual organization index (POI), and processing speed index (PSI).

### Image Acquisition

MR imaging was performed with 3-Tesla PET/MR scanner (SIGNA PET/MR; General Electric Medical Systems, Milwaukee, WI, United States) with an 8-channel head coil. High-resolution T1-weighted anatomical MRI (repetition time (TR) = 6.38 ms; echo time (TE) = 1.99 ms; flip angle (FA) = 11°; field of view (FOV) = 256 mm; 256 × 256 matrix; 172 slices; voxel dimension = 1.0 mm × 1.0 mm × 1.0 mm) were collected at the University of Fukui Hospital. Diffusion weighted images were acquired using single-shot echo-planar imaging (EPI) (acquisition matrix = 128 × 128; TE = Minimum; TR = 9327 msec; FOV = 240 mm; 240 × 240 matrix; pixel size = 1.9 mm^2^ × 1.9 mm^2^; 45 axial slices; slice thickness/gap = 3.0 mm/0 mm) with 30 distributed isotropic orientations for the diffusion-sensitizing gradients at a *b*-value of 1000 s/mm2 and a *b*-value of 0.

### Image Analysis

We used Freesurfer 6.0^1^ to reconstruct each participant’s T1-weighted anatomical image (Dale et al., 1999) and obtain gray and WM volumes and cortical and subcortical regions in individual subjects. The diffusion data were submitted to analysis using TRActs Constrained by UnderLying Anatomy (TRACULA; Yendiki et al., 2011), an automated global probabilistic tractography tool in Freesurfer, which delineates 18 WM pathways in the participants’ DWI data. The method relies on prior knowledge of pathway anatomy to reconstruct the tracts based on a manually labeled training set of subjects. The pathway reconstruction is calculated separately for every point along the trajectory of the pathway and each anatomical segmentation label across the gray/WM surface obtained from the T1-weighted images using segmentation. For tensor estimation, a ball-and-stick model of diffusion was used to extract anisotropy and diffusivity measures. To ensure data quality, all images were assessed for major artifacts at the outset and excluded as necessary. To quantify head motion in each participant’s DWI data, we calculated four measures of head motion: translation (mm), rotation (degree), average signal intensity drop out score, and portion of slices with greater than the computed drop-out score (%), and removed eddy current-induced image distortions (Yendiki et al., 2014).

**FIGURE 1:**
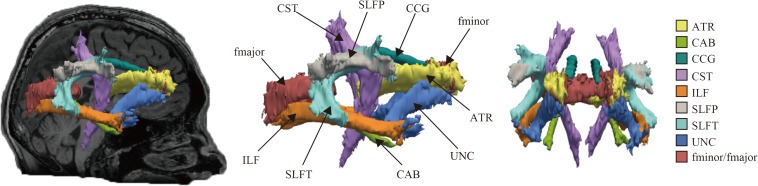
Probabilistic reconstruction of white matter pathways. Reconstructed white matter (WM) pathways in study participants, displayed at 20% of their maximum threshold on selected slices of T1 image in a study individual using the global probabilistic algorithm proposed in TRACULA software. ATR, anterior thalamic radiation; CAB, cingulum-angular bundle; CCG, cingulum-cingulate gyrus bundle; CST, corticospinal tract; ILF, inferior longitudinal fasciculus; SLFP, superior longitudinal fasciculus-parietal ending; SLFT, superior longitudinal fasciculus-temporal ending; UNC, unicate fasciculus; fmajor, corpus callosum-forceps major; fminor, corpus callosum forceps minor.

The reconstructed pathways consisted of Figure 1: anterior thalamic radiations (ATR), cingulum-angular bundle (CAB), CCG, corticospinal tract (CST), inferior longitudinal fasciculus (ILF), superior longitudinal fasciculus-parietal terminations (SLFP), superior longitudinal fasciculus-temporal terminations (SLFT), uncinate fasciculus (UNC), corpus callosum-forceps major (fmajor), and corpus callosum-forceps minor (fminor).

Finally, we extracted four diffusion measures (FA, MD, RD, and AD) at each voxel, weighted by the pathway probability in each of the 18 WM pathways by thresholding the pathway distribution at 20% of its maximum value for each participant.

### Statistical Analyses

Demographic, IQ, and DTI scalars of WM pathways were submitted to statistical analysis (SPSS, version 22; IBM Corporation) to examine sex differences in the neural mechanisms of language. In keeping with previous studies (Vos et al., 2011; Pfuhl et al., 2016), we used analysis of covariance (groups and pathways) for each diffusion measure (FA, MD, RD, and AD) with tract volume and age as nuisance regressors.

Considering the possible influence of age and tract volume on the correlation analysis between WM measures and language ability, we performed partial correlation analysis, controlling for the effects of age and tract volume, to examine the strength of the relationship between the diffusion measures and language ability (VIQ, VC, and WMI), and performance ability (PIQ, POI, and PSI) for each WM pathway separately in each group (male and female). We calculated both Pearson’s correlation and Spearman’s correlation coefficients (parametric and non-parametric analyses) for each group to confirm the findings. The level of statistical significance for each diffusion measure was corrected using a false discovery rate (FDR) of *q* < 0.05 across all pathways simultaneously. Correlations between language ability and WM pathway measures (r1 and r2) implicated in either group (males and females; n1, n2 group sizes) were then tested for group difference to assess sex differences in WM pathways related to language ability following the formula (Lowry, 2014).

$$z=\frac{\left(\frac{1}{2}\,\ln\frac{1-r_{1}}{1-r_{1}}\right)-\left(\frac{1}{2}\,\ln\frac{1-r_{2}}{1-r_{2}}\right)}{\sqrt{\frac{1}{n_{1}-3}+\frac{1}{n_{2}-3}}}$$

## Results

### Participant Test Scores

The cognitive profile of the study participants is presented in Table 1. There were no significant differences between the male and female groups for age (*p* = 0.53), FSIQ (*p* = 0.82), VIQ (*p* = 0.89), and PIQ (*p* = 0.98). Moreover, there were no significant differences between the two groups for VCI (*p* = 0.47), WMI (*p* = 0.10), POI (*p* = 0.66), and PSI (*p* = 0.40).

**TABLE 1:** Cognitive profiles of male and female subjects.

	**Male**	**Female**	***p*-value**
**Measure**	**(*n* = 32)**	**(*n* = 35)**	
Full scale IQ (SD)	114.0 (8.8)	113.4 (8.4)	0.80
Verbal IQ (SD)	115.6 (10.5)	116.1 (16.0)	0.89
Performance IQ (SD)	110.1 (12.3)	110.1 (10.8)	0.98
Subtest index			
Verbal comprehension index (SD)	112.9 (10.8)	111.1 (9.5)	0.47
Working memory index (SD)	112.3 (11.3)	108.1 (9.3)	0.10
Perceptual organization index (SD)	108.6 (11.6)	107.3 (11.9)	0.66
Processing speed index (SD)	112.3 (11.2)	114.7 (12.6)	0.40

*SD, standard deviation in parentheses.*

### Sex Differences in WM Pathways

**FIGURE 2:**
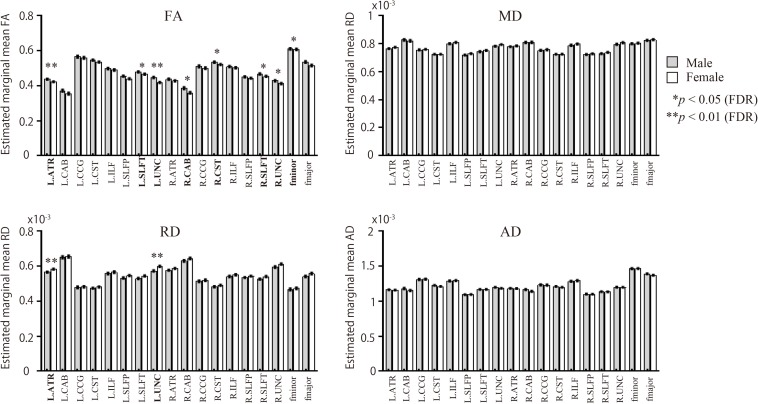
Sex differences in WM pathways. Bar graphs depict estimated marginal means for FA, MD, RD, and AD for each of the 18 pathways. The gray bars represent male participants. The white bars represent female participants. FA, fractional anisotropy; MD, mean diffusivity; AD, axial diffusivity; RD, radial diffusivity. Comparison of diffusion measures between male and female groups for the 18 pathways were based on univariate analyses using ANCOVA, covarying for age and volume of each pathway; *p*-values were calculated following adjustments for age and each pathway’s volume.

Male participants showed higher FA in the left ATR, right CAB, right CST, bilateral SLFT, bilateral UNC, and fminor when compared with the female group (Figure 2 and Table 2). There were no WM tracts with FA values that were lower in the males than in the females. The female group, however, showed higher RD in the left ATR and left UNC when compared to the male group. There were no WM pathways with RD values lower in the females than in the males. There were no statistically significant differences between the groups in the MD or AD of any of the reconstructed paths.

**TABLE 2:** White matter metrics of pathways reconstructed using Tracula.

	**FA**				**MD**				**RD**				**AD**			
		**Effect**	**Observed**			**Effect**	**Observed**			**Effect**	**Observed**			**Effect**	**Observed**	
	***p*-value**	**size**	**power**	**FDR**	***p*-value**	**size**	**power**	**FDR**	***p*-value**	**size**	**power**	**FDR**	***p*-value**	**size**	**power**	**FDR**
L.ATR	0.001	0.154	0.915	0.009	0.012	0.095	0.717	0.117	0.000	0.216	0.984	0.000	0.614	0.004	0.079	1.000
R.ATR	0.123	0.037	0.337	0.170	0.097	0.043	0.382	0.249	0.029	0.073	0.595	0.065	0.859	0.001	0.054	1.000
L.CAB	0.042	0.064	0.534	0.069	0.393	0.012	0.135	0.590	0.749	0.002	0.062	0.749	0.036	0.068	0.558	0.231
R.CAB	0.002	0.144	0.893	0.012	0.853	0.001	0.054	0.903	0.061	0.054	0.466	0.122	0.043	0.064	0.531	0.231
L.CCG	0.466	0.008	0.112	0.493	0.452	0.009	0.116	0.626	0.624	0.004	0.077	0.661	0.749	0.002	0.062	1.000
R.CCG	0.197	0.026	0.251	0.253	0.568	0.005	0.087	0.682	0.427	0.010	0.123	0.512	0.922	0.000	0.051	1.000
L.CST	0.031	0.071	0.583	0.056	1.000	0.000	0.050	1.000	0.098	0.043	0.380	0.160	0.071	0.051	0.441	0.231
R.CST	0.024	0.078	0.625	0.048	0.708	0.002	0.066	0.797	0.125	0.037	0.334	0.173	0.077	0.049	0.426	0.231
L.ILF	0.285	0.018	0.186	0.321	0.224	0.023	0.227	0.448	0.271	0.019	0.194	0.348	0.599	0.004	0.081	1.000
R.ILF	0.281	0.018	0.188	0.321	0.144	0.034	0.307	0.324	0.117	0.038	0.346	0.173	0.523	0.007	0.097	1.000
LSLFP	0.008	0.106	0.766	0.030	0.041	0.064	0.536	0.246	0.012	0.096	0.721	0.059	0.720	0.002	0.065	1.000
R.SLFP	0.094	0.044	0.388	0.141	0.303	0.017	0.176	0.545	0.096	0.043	0.384	0.160	0.890	0.000	0.052	1.000
L.SLFT	0.016	0.089	0.684	0.036	0.081	0.048	0.416	0.249	0.022	0.081	0.640	0.064	1.000	0.000	0.050	1.000
R.SLFT	0.010	0.101	0.743	0.030	0.091	0.045	0.394	0.249	0.017	0.087	0.676	0.061	0.738	0.002	0.063	1.000
L.UNC	0.000	0.315	1.000	0.000	0.013	0.094	0.712	0.117	0.000	0.250	0.995	0.000	0.061	0.055	0.468	0.231
R.UNC	0.014	0.092	0.703	0.036	0.057	0.056	0.481	0.249	0.013	0.094	0.709	0.059	0.862	0.000	0.053	1.000
fminor	0.009	0.104	0.759	0.030	0.347	0.014	0.154	0.568	0.025	0.077	0.619	0.064	0.049	0.060	0.508	0.231
fmajor	0.668	0.003	0.071	0.668	0.508	0.007	0.100	0.653	0.471	0.008	0.110	0.530	1.000	0.000	0.050	1.000

*ATR, anterior thalamic radiation; CAB, cingulum-angular bundle; CCG, cingulum-cingulate gyrus bundle; CST, corticospinal tract; ILF, inferior longitudinal fasciculus; SLFP, superior longitudinal fasciculus-parietal ending; SLFT, superior longitudinal fasciculus-temporal ending; UNC, unicate fasciculus; fmajor, corpus callosum-forceps major;fminor, corpus callosum forceps minor.*

### Associations Between Language Ability and WM Pathways

**FIGURE 3:**
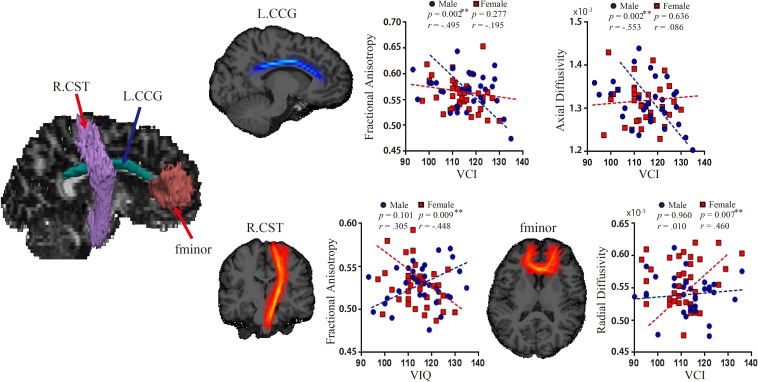
Relationship between WM pathways and language ability. Left, white matter pathways revealed significant associations with language. Right (top row), significant associations between VCI and FA (*r* = –0.495, *p* = 0.002), and between VCI and AD (*r* = –0.553, *p* = 0.002) in left CCG in the male group. Right (bottom row), significant associations between VIQ and FA in the right CST (*r* = –0.448, *p* = 0.009), and between VCI and RD in the fminor (*r* = 0.460, *p* = 0.007) in the female group. CCG, cingulum-cingulate gyrus supracallosal bundle; CST, corticospinal tract; FA, fractional anisotropy; RD, radial diffusivity; VCI, verbal comprehension index; VIQ, verbal IQ.

There were significant negative relationships between VCI and FA (Pearson’s *r* = −0.495, *p* = 0.002; Spearman’s *r* = −0.434 *p* = 0.014), and between VCI and AD (Pearson’s *r* = −0.553, *p* = 0.002; Spearman’s *r* = −0.501 *p* = 0.004) in left CCG in the male group (Figure 3). No other associations between WM metrics and VIQ or WMI in the male group were significant. In contrast, the female group showed a significant negative relationship between VIQ and FA in the right CST (Pearson’s *r* = −0.448, *p* = 0.009; Spearman’s *r* = −0.409, *p* = 0.018), and a positive one between VCI and RD in the fminor (Pearson’s *r* = 0.460, *p* = 0.007; Spearman’s *r* = 0.293, *p* = 0.097). There were no significant associations between WMI and WM pathways in the female group. Moreover, there were sex differences between the correlation coefficients of the two groups for the VCI – AD association in the left CCG (*z* = 2.77, *p* = 0.0056), and for VIQ – FA association in the right CST (*z* = 3.11, *p* = 0.0019).

### Associations Between Performance Ability and WM Pathways

The male group showed significant positive relationship between POI and AD in the left ILF (Pearson’s *r* = 0.501, *p* = 0.005; Spearman’s *r* = 0.505, *p* = 0.004). There were no significant associations between PIQ or PSI, and WMI pathways in the male group. In contrast, the female group showed significant negative relationship between PIQ and FA in the right CAB (Pearson’s *r* = −0.505, *p* = 0.003; Spearman’s *r* = −0.389, *p* = 0.025). There were no significant relationships between POI, or PSI, and WM pathways in the female group. Moreover, there were sex differences between two correlation coefficients of the two groups for the POI – AD association in the left ILF (*z* = 2.71, *p* = 0.0067), and PIQ – FA association in the right CAB (*z* = 2.98, *p* = 0.0029).

## Discussion

The present study found significant sex differences in WM pathways related to language. Adult males displayed higher FA across multiple WM pathways when compared to adult females, whereas the females tended to have higher RD in some of the pathways. With regard to language ability, male participants showed a relationship between language comprehension and FA as well as AD of the CCG, whereas the female group showed a significant relationship between VIQ and FA in the CST and between language comprehension and RD in fminor. Further analyses revealed a significant difference between correlation coefficients of the two groups for the VCI-AD association in left CCG and for VIQ-FA association in right CST. These findings highlight the sex differences in WM pathways related to language ability.

Consistent with previous studies (Lebel et al., 2010; Clayden et al., 2012), we found that males demonstrated higher FA in WM pathways when compared with females. These sex differences have been found in WM microstructure (Kanaan et al., 2012), WM volume (Gur et al., 1999) and WM pathways (Lebel et al., 2010; Clayden et al., 2012). However, we found that the female group demonstrated higher RD of the tracts when compared with the males, similar to findings in other studies (Kumar et al., 2013). These findings converge with previous diffusion tensor imaging (DTI) results (Song et al., 2005; Bar-Shir et al., 2009; Wheeler-Kingshott and Cercignani, 2009; Billiet et al., 2015; Boord et al., 2015) suggesting that myelination plays a pivotal role in anisotropy and diffusivity changes. More specifically, FA is associated with the amount of myelination (Wheeler-Kingshott and Cercignani, 2009) whereas RD is associated with the degree of myelination (Song et al., 2005). Taken together, these findings may reflect not only sex differences of WM pathways, but also the changes in myelin content during maturation.

In the current study, FA and AD in the CCG were negatively associated with verbal comprehension but only in the male group. Previous studies have revealed sex differences in verbal memory tasks (Bleecker et al., 1988; Kramer et al., 1997; Volf and Razumnikova, 1999; Lewin et al., 2001; Hill et al., 2006). For example, males show an advantage in visuospatial episodic memory and spatial word memory during language use, whereas a female advantage is evident in verbal episodic memory tasks (Lewin et al., 2001; Pruden and Levine, 2017). Perhaps, then, our findings may be due to the use of different coding strategies (viz., visuospatial vs. verbal) in males compared to females, while also reflecting a sex difference in the neural mechanism of language evident in verbal comprehension. A key role for FA in the CCG has also been suggested previously in a DTI study of episodic memory using a visual cue reminder task (Ezzati et al., 2016). FA also represents an overall measure of fiber density (Song et al., 2005) and numerous fibers join and leave the CCG bundle, adding to the complexity of isolating pathways and contributions of this complex tract. When taken together, however, our results suggest that FA and AD parameters may serve as valuable measures of sex differences in the neural mechanism of language related to fiber density.

A meta-analysis of functional imaging studies revealed sex differences in verbal fluency and speech production (Sommer et al., 2004). Other studies, using DTI, have suggested that speech production is associated with FA in the CST (Tokimura et al., 1996; Lin et al., 2002; Welniarz et al., 2017). In the present study, we found a negative association between verbal ability and FA in the CST in females. We also found a positive association only in the female group between verbal comprehension ability and RD in the fminor which is known to play a central role in integration of higher level language (Solso et al., 2016). The fminor pathway is not only associated with second language proficiency (Nichols and Joanisse, 2016) but also sex differences (Wang et al., 2012). Taken together, the fminor-associated finding in our female group may reflect a sex difference in language proficiency and the neural basis of it.

Some of our analyses revealed significant negative correlations between FA and verbal performance scores. Similar negative correlations between FA and verbal ability have been reported using regression analysis (Konrad et al., 2012; Madhavan et al., 2014). These previous studies have suggested that an increasing or decreasing FA may be related to the language measure being used, such as VIQ (Konrad et al., 2012) which differs from verbal fluency (Madhavan et al., 2014). Taken together with these findings, our study provides complementary support of the notion that the relationship between FA and language ability is not always a positive association but may depend on the type of language ability being tapped.

The present study found an association between AD in the ILF and perceptual organization but only in males. The ILF is WM pathway with long and short fibers connecting the visual area and limbic areas, such as the amygdala and hippocampus. The ILF is involved not only in language (Catani and Thiebaut de Schotten, 2008), but also in visual perception (ffytche, 2008), place perception (Hodgetts et al., 2015), and object recognition (Ortibus et al., 2012). A previous study showed that the degree of AD may be affected by factors including the number of axons, axonal diameter and aging in autism spectrum disorder, a condition known to be more prevalent in males than in females (Karahanoğlu et al., 2018). Additionally, WM integrity in the ventral temporal lobe has been associated with superior visuospatial perception in individuals with ASD (Sahyoun et al., 2010; Jung et al., 2019). As such, these findings lend support to the interpretation, that the association between AD in the ILF and POI only in the male group may reflect a basis for the sex difference in the neural mechanism related to perceptual ability.

The male and female groups in this study did not differ on any of the cognitive measures, including language ability. As such, we focused on exploring the correlations between diffusion measures and verbal ability measures separately for each tract in each group and then testing the significant correlations for difference between the male and female groups (i.e., a sex difference). TRACULA uses global probabilistic tractography and yields only 18 pathways which we examined for WM associations with language ability in each group. We found a significant group difference in FA of the SLFT (also known as the arcuate fasciculus), a tract which has been implicated in language (Middlebrooks et al., 2017), but it did not survive FDR correction. Finally, the WAIS-III although widely used, is a relatively broad measure of verbal ability. The current version of the test (WAIS-IV) was not yet available when data collection started. Using a measure that specifically taps language processes would have allowed for a better characterization of the neural mechanism of sex differences related to language. It would be important to address these weaknesses in future studies. Furthermore, a test measure specifically designed to tap language processes would have allowed for better characterization of the neural mechanism of sex differences related to language. It would be important to address these weaknesses in future studies.

## Conclusion

In conclusion, male adults displayed higher FA, whereas female adults displayed higher RD across WM pathways in the current study. Moreover, our results suggest a role for CCG and CST in the neural basis of sex differences in language. These findings shed light on the influence of sex on the neural mechanism of language.

## Data Availability

The datasets generated for this study are available on request to the corresponding author.

## Ethics Statement

Human Subject Research: The studies involving human participants were reviewed and approved by the ethics committee of the University of Fukui. The patients and participants provided their written informed consent to participate in this study.

## Author Contributions

MJ was involved in conducting the experiment, analyzing and interpreting the data, and drafting the manuscript. MM was involved in designing the analysis, interpreting the data, and drafting the manuscript. TF, YK, and HO were involved in recruiting the participants and drafting the manuscript. HK was involved in diagnosing the participants, conducting the experiment, analyzing and interpreting the data, and drafting the manuscript.

## Conflict of Interest Statement

The authors declare that the research was conducted in the absence of any commercial or financial relationships that could be construed as a potential conflict of interest.

## Footnotes

1 http://surfer.nmr.mgh.harvard.edu
